# Assessment of the diagnostic value of diffusion tensor imaging in patients with spinal cord compression: a meta-analysis

**DOI:** 10.1590/1414-431X20154769

**Published:** 2015-11-27

**Authors:** X.F. Li, Y. Yang, C.B. Lin, F.R. Xie, W.G. Liang

**Affiliations:** Department of Spine Surgery, Guangxi Orthopaedics and Traumatology Hospital, Nanning, China

**Keywords:** Diffusion tensor imaging, Apparent diffusion coefficient, Fractional anisotropy, Spinal cord compression, Meta-analysis

## Abstract

We investigated the diagnostic value of the apparent diffusion coefficient (ADC) and fractional anisotropy (FA) of magnetic resonance diffusion tensor imaging (DTI) in patients with spinal cord compression (SCC) using a meta-analysis framework. Multiple scientific literature databases were exhaustively searched to identify articles relevant to this study. Mean values and standardized mean differences (SMDs) were calculated for the ADC and FA in normal and diseased tissues. The STATA version 12.0 software was used for statistical analysis. Of the 41 articles initially retrieved through database searches, 11 case-control studies were eligible for the meta-analysis and contained a combined total of 645 human subjects (394 patients with SCC and 251 healthy controls). All 11 studies reported data on FA, and 9 contained data related to the ADC. The combined SMDs of the ADC and FA showed that the ADC was significantly higher and the FA was lower in patients with SCC than in healthy controls. Subgroup analysis based on the b value showed higher ADCs in patients with SCC than in healthy controls at b values of both ≤500 and >500 s/mm^2^. In summary, the main findings of this meta-analysis revealed an increased ADC and decreased FA in patients with SCC, indicating that DTI is an important diagnostic imaging tool to assess patients suspected to have SCC.

## Introduction

Spinal cord compression (SCC) is generally described as any condition that places abnormal pressure on the spinal cord. Increased pressure can be caused by bone fragments from vertebral fractures, abscesses, rheumatoid arthritis, infection, tumors, ruptured intervertebral discs, spinal malformations, or other lesions (1). SCC can precipitate immediately, such as in impact injuries, or take months or years to develop, such as in tumors or secondary to wear and tear of the spine. Generally, the most common symptoms of SCC are back pain, limb paralysis, sensory loss, urinary and fecal incontinence or urinary retention, sphincter dysfunction, sexual dysfunction, autonomic nervous system dysfunction, and loss of spinal cord function (2,3). SCC is clinically present in 5-14% of patients with cancer during the progression of their malignancy, and 2-5% of patients with cancer have at least one episode of compression within their final 2 years of life (4). In England and Wales, the annual prevalence of SCC is 80 per 1,000,000 persons, which is equivalent to 4000 cases each year (5). Pain, either local or radicular, is the presenting symptom in approximately 90-95% of patients with SCC, and more than 50% of patients with SCC may lose their ability to walk and exhibit sensory disturbances or autonomic dysfunction (3). Although treatment approaches for SCC have improved over the years, the condition continues to have a substantial negative impact on the quality of life of patients and their families (6). Therefore, early diagnosis of SCC is very important for timely and appropriate treatment and improved outcomes. In this context, diffusion tensor imaging (DTI) has recently been introduced as a diagnostic approach for SCC (7).

DTI is an advanced magnetic resonance (MR) imaging method to evaluate the micro-structural architecture of the brain by measuring the three-dimensional shape and direction of diffusion via the apparent diffusion coefficient (ADC) and fractional anisotropy (FA) (8,9). The ADC represents the magnitude of diffusion and reflects the capacity of molecular water to diffuse in different directions in a three-dimensional tissue space, while FA assesses the orientation coherence (or anisotropy) of diffusion and provides valuable information on white matter fiber integrity (10,11). FA values range from 0 (isotropic; i.e., diffusion is uniform and equal in all directions) to 1 (purely anisotropic; i.e., absolutely in one direction) and increase with age and tissue maturity (12). There are four ways to obtain imaging sequences for diffusion-weighted imaging (DWI), and thereby for DTI. These include single-shot echo-planar imaging (SS-EPI), sagittal spin-echo SS-EPI (SE-SS-EPI), single-shot fast spin-echo (SS-FSE), and FSE. SS-EPI has low sensitivity to motion-mediated phase errors that occur in diffusion sensitization of the MR signal, but has limited spatial resolution (13). SE-SS-EPI is characterized by fast acquisition times, insensitivity to subject motion, and a comparatively high image resolution (1). The spatial resolution and acquisition time of SS-FSE are identical to those of SE-SS-EPI, although multiple images can be acquired with SS-FSE (14). FSE is very fast and has high image quality, sensitivity, and T2-contrast (15). The diagnostic value of DTI has been examined in various clinical settings, such as in traumatic brain and spinal cord injuries, tumor resection, and stroke (16,17). Recent studies have shown that the ADC is increased and that FA is decreased in patients with SCC, suggesting that DTI may be helpful in the diagnosis of SCC (1,7). Low FA reflects damage to tissue structures and corresponds to pathological changes such as demyelination or dysmyelination, axonal loss, gliosis, cyst formation, and necrosis (18-20). SCC may lead to cell death or increased cell membrane permeability, resulting in extracellular edema (21). Furthermore, SCC may induce abnormal flow of cerebrospinal fluid to the inner part of the spinal cord, causing the formation of small cysts within the spinal cord (22). The two conditions described above also increase the ADC and decrease FA values; therefore, a possibility exists that these parameters could be used for diagnosis (7).

Multiple previous studies have shown that DTI may be a reliable early diagnostic method for SCC and that it can guide clinical treatment choices for patients with SCC (23,24). However, other studies found contrasting results (21,25). In this meta-analysis, we examined the diagnostic value of DTI in assessing SCC and its potential as an early diagnostic tool for SCC.

## Material and Methods

### Literature search and data sources

Our meta-analysis conformed to PRISMA reporting standards. A comprehensive online search was performed to identify relevant articles archived in the following databases: EMBASE (1999-2014), CINAHL (1999-2014), Cochrane (2005-2014), PubMed (1966-2014), ISI Web of Science (1990-2014), Chinese National Knowledge Infrastructure (1990-2014), WANFANG DATA (1990-2014), and CQVIP (1990-2014). The last search conducted was in April 2014. The search terms related to DTI and SCC were (“Spinal Cord Compression,” “conus medullaris syndrome,” “malignant spinal cord compression,” “epidural spinal cord compression,” “metastatic spinal cord compression,” “extradural spinal cord compression,” “malignant epidural spinal cord compression,” “MESCC,” “neoplastic spinal cord compression,” “MSCC”) and (“Diffusion Tensor Imaging,” “diffusion tensor imaging,” “imaging, diffusion tensor,” “diffusion tractography,” “DTI”). The search had no language restrictions, and language translation was conducted as necessary. All retrieved articles were carefully screened, and full texts of the screened articles were obtained. Additional articles were manually identified from the reference lists of selected articles and reviewed for their relevance to this study.

### Inclusion and exclusion criteria

The included studies met the following criteria: 1) the study design was a case-control study; 2) DTI was used to distinguish patients with SCC from a healthy control group; 3) the minimum number of patients in the study was five and the minimum number of diffusion-encoding directions was six to provide sufficient reliability of study results; 4) the standard diagnostic criteria for SCC were motor dysfunction, neck pain, sensory deficits for more than 6 weeks, compressive lesions (cervical spondylotic myelopathy, ossification of the ligament flavum, ossification of the posterior longitudinal ligament, and cervical spinal stenosis), and cervical disc herniation as visualized on MR imaging; 5) the largest relevant sample size was chosen if more than two overlapping patient samples existed; 6) the study was performed on humans and published in a peer-reviewed journal; and 7) the article provided original data and sufficient information on the ADC and/or FA in patients with SCC and healthy subjects. The exclusion criteria were 1) reviews, abstracts, letters, non-human studies, non-case-control studies, or duplicate studies; 2) studies not related to the research topic; 3) studies with incomplete data; 4) an unclear diagnosis for the study subjects; and 5) non-English or non-Chinese publications. Only the study with the largest sample size or the latest study was included when the selected studies were published by the same authors with the same case materials.

### Study quality and data extraction

The studies were independently assessed by two reviewers based on the inclusion/exclusion criteria. Disagreement on inclusion of any single study was resolved by discussion or consultation with a third investigator. A standard data form was used to collect the following study information: surname and initials of the first author, publication year or submission year, source country and ethnicity, language of publication, sample size, demographic variables of the subjects, type of technique for DWI, MR imaging machine type, MR imaging machine type code, b value (s/mm^2^), ADC (×10^-3^mm^2^/s), and FA value in normal subjects and patients. Each full-text article was scored using the Quality Assessment of Diagnostic Accuracy Studies (QUADAS) tool (26). The following items were scored. QUADAS01: Was the spectrum of patients representative of various disease spectrums? QUADAS02: Were the selection criteria for the subjects clearly described? QUADAS03: Was the time period between the reference standard and detection test short enough? QUADAS04: Did the whole sample receive verification using a reference standard of diagnosis? QUADAS05: Did patients receive the same reference standard? QUADAS06: Was the reference standard independent of the index test? QUADAS07: Were the index test results judged without knowledge of the results of the reference standard? QUADAS08: Were the results of the reference standard judged without knowledge of the index test results? QUADAS09: Were the same clinical data available when the results were interpreted and the test was used in practice? QUADAS10: Were uninterpretable or intermediate test results reported? QUADAS11: Were patients outside of the study explained?

### Statistical analysis

The mean and standardized mean difference (SMD) of the ADC and FA in normal and diseased tissues were calculated. The Z test was used to calculate the 95% confidence interval (95%CI). Cochran’s *Q*-statistic and *I2* tests were used to calculate heterogeneity between samples (27). A subgroup analysis was performed if substantial heterogeneity was found in the ADC and FA values (and 95%CI) in the patients with SCC. A one-way sensitivity analysis was employed to evaluate the effect of single studies on the overall estimate. Furthermore, the potential publication bias was detected by Egger’s linear regression test with a funnel plot for visual inspection (28,29). STATA statistical software (Version 12.0; Stata Corporation, USA) was used for statistical analysis.

## Results

### Included studies

**Figure 1:**
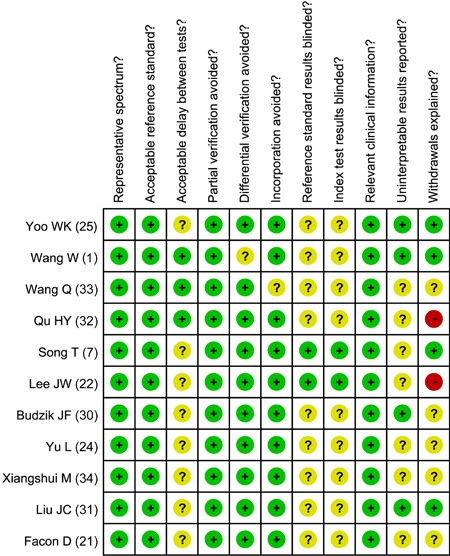
Methodological quality assessment for each of the included case-control studies on the basis of the Quality Assessment of Diagnostic Accuracy Studies (QUADAS) tool.

Both electronic database and manual searches were employed, which resulted in identification of 41 potentially relevant articles. After a cursory review of titles and abstracts, three duplicate studies were removed and 11 irrelevant articles were excluded because they were irrelevant article types, non-human studies, or had no relation to the research topic. The remaining 27 articles were carefully reviewed by full-text reviews, and only 12 articles contained suitable qualitative analysis; the other 15 articles were not case-control studies or were not related to DTI or SCC and thus were eliminated. Among the 12 studies with suitable qualitative analysis, one study was excluded because of lack of data integrity after a more thorough assessment of the study. Finally, 11 case-control studies containing 645 human subjects (394 patients with SCC and 251 healthy controls) were selected for the current meta-analysis (1,7,21 22 23 24 25,30 31 32 33 34). The sample sizes in these 11 studies ranged from 11 to 105 human subjects. The publication year of the studies ranged from 2002 to 2014. Nine studies were conducted in Asian populations, 7 in China, and 2 in South Korea; two studies involved Caucasians (both performed in France). Two studies (33,34) lacked age and sex information. The MR imaging machine types used in the studies included Philips 1.5/3.0T scanners and GE 1.5 T/3.0T MRI scanners. ADC (×10^-3^ mm^2^/s) and FA values in the patients with SCC and the healthy controls were reported as mean±SD. The baseline characteristics of individual studies and the ADC and FA values are shown in Supplementary Table S1. The methodological quality assessment of each individual study on the basis of QUADAS is presented in Figure 1.

### Quantitative data synthesis

**Figure 2:**
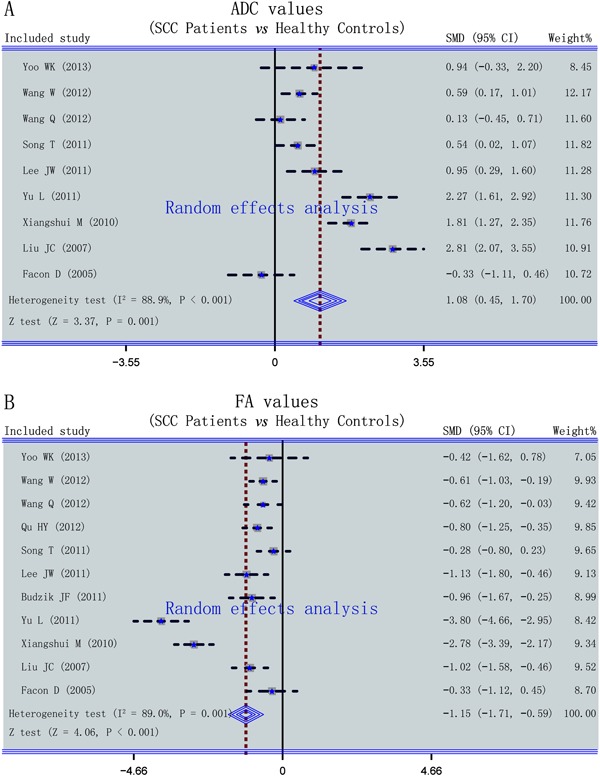
Forest plots of the difference in the frequency of the apparent diffusion coefficient (ADC) and fractional anisotropy (FA) values between patients with spinal cord compression (SCC) and healthy subjects (*A*, ADC; *B*, FA value). See Figure 1 for reference details. SMD: standard mean difference.

**Figure 3:**
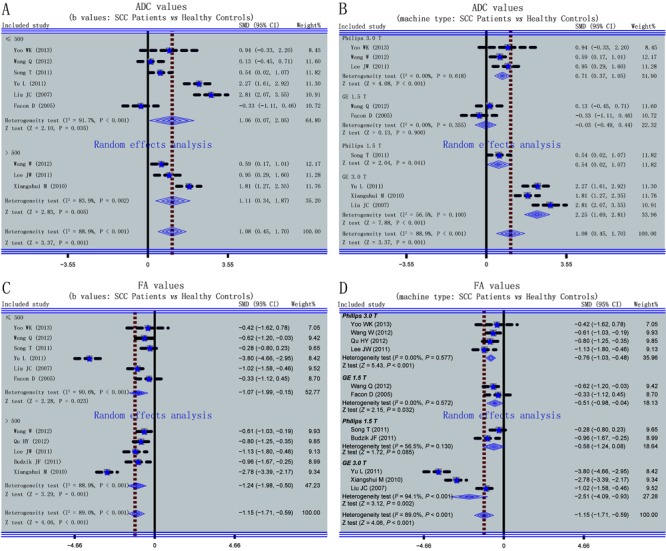
Subgroup analyses of the difference in the apparent diffusion coefficient (ADC) and fractional anisotropy (FA) values between patients with spinal cord compression (SCC) and healthy subjects (*A*, ADC b value; *B*, ADC machine type; *C*, FA b value; *D*, FA machine type). See Figure 1 for reference details. SMD: standard mean difference.

Nine studies reported the ADC, and a random-effects model was chosen because of the presence of heterogeneity among the studies as indicated by the *Q*-test and *I2* test (all *I2*=88.9%, P<0.001). Significant differences in the ADC were detected between patients with SCC and healthy controls in this meta-analysis. The ADC was significantly higher in patients with SCC than in healthy controls according to the random effects incorporation of the pooled SMD of the 11 studies (SMD=1.08, 95%CI=0.45-1.70, P*=*0.001) (Figure 2A). Stratified studies based on the b value and MR imaging machine type were performed to explore the potential sources of heterogeneity among the studies. The b-value stratified analysis showed a higher ADC in patients with SCC than in healthy controls at b values of both ≤500 and >500 (b value ≤500: SMD=1.06, 95%CI=0.07-2.05, P*=*0.035; b value >500: SMD=1.11, 95%CI=0.34-1.87, P*=*0.005) (Figure 3A). The MR imaging machine type-stratified analysis revealed significantly higher ADCs in patients with SCC than in healthy controls using the following MR imaging machine type subgroups: Philips 3.0/1.5 T and GE 3.0 T (Philips 3.0 T: SMD=0.71, 95%CI=0.37-1.05, P*<*0.001; Philips 1.5 T: SMD=0.54, 95%CI=0.02-1.07, P*=*0.041; GE 3.0 T: SMD=2.25, 95%CI=1.69-2.81, P*<*0.001), but not using the GE 1.5 T (SMD=-0.03, 95%CI=-0.49 to 0.44, P*=*0.900) (Figure 3B).

Eleven studies reported FA values, and the heterogeneity test indicated significant heterogeneity (*I2*=89.0%, P=0.001); thus, a random-effects model was applied. The main result of this meta-analysis showed that the FA values were markedly lower in patients with SCC than in healthy controls (SMD=-1.15, 95%CI=-1.71 to 0.59, P<0.001) (Figure 2B). Subgroup analysis based on the b value demonstrated significantly lower FA values in patients with SCC than in healthy controls at b values of both ≤500 and >500 (b value ≤500: SMD=-1.07, 95%CI=-1.99 to 0.15, P=0.023; b value >500: SMD=-1.24, 95%CI=-1.98 to 0.50, P=0.001) (Figure 3C). Further subgroup analysis based on MR imaging machine types showed lower FA values in patients with SCC than in healthy controls using the Philips 3.0 T, GE 1.5 T, and GE 3.0 T (Philips 3.0 T: SMD=-0.76, 95%CI=-1.03 to 0.48, P<0.001; GE 1.5 T: SMD=-0.51, 95%CI=-0.98 to 0.04, P=0.032; GE 3.0 T: SMD=-2.51, 95%CI=-4.09 to 0.93, P=0.002), but not using the Philips 1.5 T (SMD=-0.58, 95%CI=-1.24 to 0.08, P=0.085) (Figure 3D).

**Figure 4:**
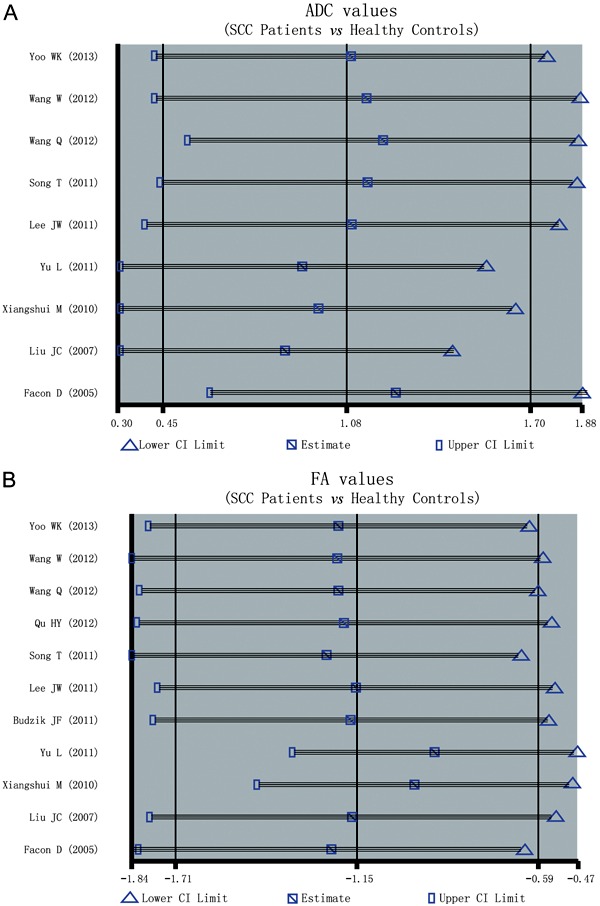
Sensitivity analysis of the summary odds ratio coefficients on the difference in the frequency of the apparent diffusion coefficient (ADC) and fractional anisotropy (FA) values between patients with spinal cord compression (SCC) and healthy subjects (*A*, ADC; *B*, FA value). See Figure 1 for reference details.

**Figure 5:**
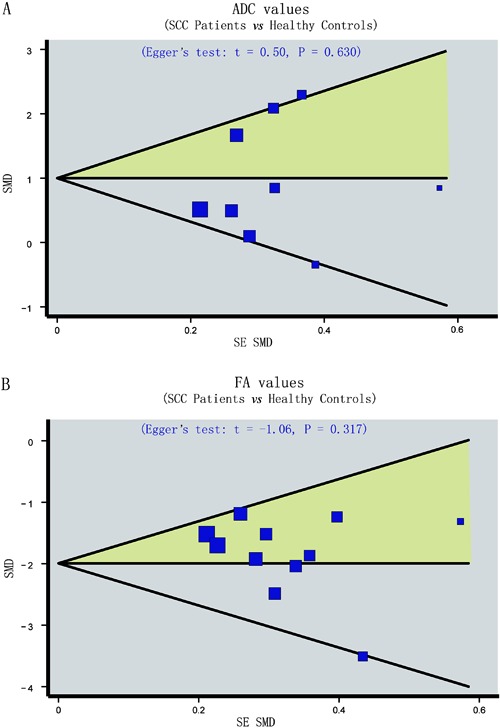
Figure 5. Funnel plot of publication biases on the difference in the frequency of the apparent diffusion coefficient (ADC) and fractional anisotropy (FA) values between patients with spinal cord compression (SCC) and healthy subjects (A, ADC; B, FA value). See Figure 1 for reference details.

Sensitivity analysis was conducted to determine if the results of this study were affected by any one single study, and the results suggested that no single study affected the pooled SMDs of the ADC (Figure 4A) or FA (Figure 4B) in the meta-analysis. Finally, Egger’s regression test showed no asymmetrical distribution in the funnel plot of the ADC (Figure 5A) and FA (Figure 5B) in normal and diseased tissues, indicating no publication bias (ADC: P*=*0.630, FA: P*=*0.317).

## Discussion

The current meta-analysis assessed the diagnostic value of DTI in detection of SCC using the ADC and FA values calculated from DTI. The main results of our meta-analysis show that the ADC is significantly higher and the FA is significantly lower in patients with than without SCC, offering significant diagnostic potential of using these differences in DTI for early detection of SCC. DTI measures the anisotropy of water diffusion in living tissues and provides more specific information on the tissue microstructure than does conventional MR imaging; it is suitable to assess the integrity of fiber tracts, indicating the high potential for DTI in detecting subtle pathology (19). In addition, DTI can improve the diagnostic capabilities in spinal cord injury by identifying the precise location and severity of the injury and damage (35). Changes in spinal cord white matter can be reliably detected by DTI due to anisotropy in the spinal cord microstructure, with high anisotropy in white matter and low anisotropy in gray matter, which can be quantified by DTI parameters and visualized by DTI maps (36). DTI is useful to examine the structural changes in neural tissues, especially for the assessment of spinal cord damage, and has been successfully applied in patients with spinal artery stroke, cervical spondylotic myelopathy, acute spinal cord injury, and SCC (10,30,37). The two most important quantitative parameters of DTI are the ADC and FA, both of which are widely used to study the development and pathologic changes in SCC (38). ADC measures the water diffusive strength and is extremely sensitive to the abnormalities typically seen in SCC; when combined with fiber tracking, the damaged areas of the spinal cord can be interrogated better than with T2-weighted imaging (21). FA, on the other hand, is a measure of tissue fabric anisotropy and represents the ratio of diffusive anisotropy to the total diffusion; low FA is a strong indicator of poorly organized fiber tracts within the cortex (25). The ADC is relevant in patients with SCC because of decreased perfusion leading to anoxemia, ischemia and injury of cellular membranes, changes in membrane permeability resulting in extracellular edema, abnormal perfusion of cerebrospinal fluid, and cystic necrosis in the spinal cord (1,39). Low FA is attributed to restriction of water molecule diffusion in SCC and the imbalance between the intracellular and extracellular space caused by intramedullary edema (20,34). We conclude from the above discussion that the diagnostic value of DTI in detection of SCC is more sensitive and accurate than MR imaging because of the increased membrane permeability and intramedullary edema in SCC, which causes abnormal water molecule diffusion. Consistent with our study, Lee et al. (22) also observed a higher ADC and lower FA in patients with SCC than in healthy subjects. Importantly, among subjects who showed normal signals on T2-weighted images, DTI detected significantly lower FA values and higher ADC values, indicating better sensitivity than conventional MR images.

Subgroup analysis stratified by country and MR imaging machine type was performed to understand the clinical value of DTI in the diagnosis of spinal cord compression. The b value-stratified subgroup analysis demonstrated a statistically significant difference in FA and ADC between patients with SCC and healthy controls at b values of both ≤500 and >500. The b value represents the measurement sensitivity to diffusion; thus, the results of the subgroup analysis based on the b value indicate the objectivity of our analysis. The subgroup analysis based on the MR imaging machine type suggested that increased ADCs in patients with SCC were detected by the Philips 3.0 T, Philips 1.5 T, and GE 3.0 T, but not by the GE 1.5 T, and decreased FA values were detected by all MR imaging machines except the Philips 1.5 T. These findings may be related to the difference between 1.5 T and 3.0 T. The rate of absorption of radiofrequency energy at 3.0 T is four-fold higher than that at 1.5 T. Di Perri et al. (40) reported that 3.0 T scanning detects the pathology in greater detail than does 1.5 T scanning, even in clinically healthy and younger populations. Our analysis results are in agreement with previous studies that showed an increased ADC and decreased FA values in patients with SCC. Thus, DTI might be a useful early diagnostic tool that provides sufficient quantitative information in patients with SCC to warrant further analysis of their individual pathology.

Our meta-analysis has potential limitations. First, among the included studies in our meta-analysis, only one used FSE and SS-FSE for DWI, which may have impacted the overall results. Second, the data extracted for the four-fold table of the diagnostic test were insufficient; thus, we could not calculate the sensitivity, specificity, and summary receiver operating characteristic. Third, the study was retrospective; therefore, the acquisition parameters and the ADC and FA values were not optimized, which may be a possible source of bias. Fourth, significant intersubject variability was present in DTI across the different regions of the brain, and several intrinsic factors such as increased variability and lower reproducibility in these regions may be closely correlated with partial volume errors within these relatively small structures. Finally, 9 of the 11 studies were performed in Asians (in China and Korea), which may have resulted in ethnicity bias and lowered the strength of the overall results.

In summary, the mean ADC is significantly higher and FA is lower in patients with SCC than in their healthy counterparts. Thus, these two DTI parameters may be excellent diagnostic tools to improve the accuracy of early detection of SCC. However, because of the limitations discussed, further clinical research with more data and larger sample sizes is necessary to confirm our prelim-inary results.

## Supplementary Material


