# Supplementary material for: Assessment of the diagnostic value of diffusion tensor imaging in patients with spinal cord compression: a meta-analysis
Source: Braz J Med Biol Res. 2015 Nov 27;49(1):e4769. doi: 10.1590/1414-431X20154769 (PMC4681415; doi:10.1590/1414-431X20154769)
Supplement: Supplementary file 1 [file 1414-431X-bjmbr-1414-431X20154769-supp4769.pdf]

Table S1. Main characteristics and methodological quality of eligible studies.

First author	Year	Country	Ethnicity	Total	Sample size		Gender (M/F)		Age (years)		MRI machine type	b value (s/mm^2)	Technique
					Case	Control	Case	Control	Case	Control			
Yoo WK (25)	2013	South Korea	Asian	11	6	5	5/1	5/0	56.0±14.1	46.6±15.2	Philips 3.0 T	500	SS-EPI
Wang W (1)	2012	China	Asian	91	42	49	20/22	21/28	48.1±6.5	36.3±5.1	Philips 3.0 T	700	SE-SS-EPI
Wang Q (33)	2012	China	Asian	65	50	15	NR	NR	NR	NR	GE 1.5 T	500	SE-SS-EPI
Qu HY (32)	2012	China	Asian	82	42	40	22/20	NR	55 (27–80)	47 (7–85)	Philips 3.0T	700	SE-SS-EPI
Song T (7)	2011	China	Asian	73	53	20	28/25	11/9	56 (47–71)	55 (46–67)	Philips 1.5 T	400	SS-FSE
Lee JW (22)	2011	South Korea	Asian	40	20	20	13/7	NR	50 (22–67)	NR	Philips 3.0 T	600	SS-EPI
Budzik JF (30)	2011	France	Caucasian	35	20	15	10/10	8/7	57 (34–78)	55 (35–73)	Philips 1.5 T	900	SS-EPI
Yu L (24)	2011	China	Asian	60	30	30	24/6	19/11	56 (32–68)	51 (28-–60)	GE 3.0 T	500	SE-SS-EPI
Xiangshui M (34)	2010	China	Asian	105	84	21	NR	12/9	NR	43 (18–60)	GE 3.0 T	1000	FSE
Liu JC (31)	2007	China	Asian	57	32	25	18/14	14/11	51 (14–73)	47 (14-–65)	GE 3.0 T	500	SE-SS-EPI
Facon D (21)	2005	France	Caucasian	26	15	11	10/5	8/3	54	37	GE 1.5 T	500	SE-SS-EPI

M: male; F: female; NR: not reported; SS-EPI: single-shot echo-planar imaging; SE-SS-EPI: spin-echo single-shot echo-planar imaging; SS-FSE: single-shot fast spin-echo; FSE: fast spin-echo.
